# Supplementary material for: The Search of a Malaria Vaccine: The Time for Modified Immuno-Potentiating Probes
Source: Vaccines (Basel). 2021 Feb 2;9(2):115. doi: 10.3390/vaccines9020115 (PMC7913233; doi:10.3390/vaccines9020115)
Supplement: Supplementary file 1 [file vaccines-09-00115-s001.pdf]

Table S1. *P. falciparum* pre-erythrocyte stages vaccine candidates under clinical trials.

Antigen	Candidate	System/Platform	Phase			Population		Producer/Place	Result (Ref.)
			I	II	III	Children	Adults		
TRAP	ME-TRAP	AdCh63 (first dose) and boost in MVA	I	IIb				UK	Cellular immunity stimulation<br>NCT00890760<br>[1]<br>Parasite charge reduction<br>NCT01623557<br>[2]
			I					Gambia	Inducing T-cell response, well tolerated<br>NCT01450293<br>NCT01635647
			I	IIb				Burkina Faso	[3-4]
*Pf*CelTOS	FMP012	Recombinant<br>AS01B	I					USA	NCT02174978<br>[5]
LSA	LSA-3-rec		I	IIa				The Netherlands	NCT00509158<br>[6]
CSP	RTS,S	Co-expressed with HBsAg, AS01			III			Burkina Faso, Ghana, Kenya, Malawi, Mozambique, Tanzania, Gabon	25%–36% approximate efficacy<br>NCT00866619<br>[7]
	Ad35 CS	CSP expressed in Type 35-Adenovirus	I					USA	Dose-dependent humoral response<br>NCT00371189<br>[8]
			Ib					Burkina Faso	Well tolerated, mild immunogenic<br>NCT01018459<br>[9 -10]
PfSPZ	Esporozoitos atenuados por radiación (RAS)	Protocol: WRAIR 2080	I	IIa				USA	Dose-dependent protection and inoculation method<br>NCT01441167 [11]<br>NCT02215707 [12]
		Protocol: VRC 314						USA	Homologous and heterologous long-lasting protection<br>NCT02015091<br>[13]
		Protocol: 14-I-N010	I					Malí	Significant protection<br>NCT01988636

					[14]
	Protocol: BSPZV1	I		Tanzania	NCT02132299
	Protocol: EGSPZV1	I		Equatorial Guinea	NCT02418962
	Chloroquine<br>mosquito bite	I		The Netherlands	Highly effective immune response NCT00442377<br>[15]<br>NCT00757887<br>[16]
PfSPZ-Cvac	Chloroquine<br>Intradermal inoculation<br>Cryo-preserved sporozoites	I		The Netherlands	Minimal immune response<br>NCT01728701<br>[17]
	Chloroquine<br>intra venous inoculation<br>Cryo-preserved sporozoites	I		Germany	Highly effective<br>NCT02115516<br>[18]
GAP	Pf GAP $p52^{-}$/$p36^{-}$	I	IIa	USA	Attenuation and favorable immune response<br>NCT01024686<br>[19]
Pfs25-EPA<br>[20-22]	Genic attenuation<br>*Pf*GAP3KO	I		USA	Safe and effective protection<br>[19]

Table S2. *P. vivax* vaccine candidate.

Target	Antigen	Candidate	System/Platform	Phase I	Phase II	Place	Result (Ref.)
TBV	*Pvs*25	*Pvs*25H	Recombinant<br>Alhydrogel	I		USA	Antibody having functional activity<br>[23]
			Recombinant<br>Montanide ISA 51	I		USA	Immunogenic having local and systemic reactogenicity<br>NCT00295581<br>[24]
Pre eritrocyte	TRAP	*Pv*TRAP	ChAd63-*Pv*TRAP/MVA-*Pv*TRAP	I			Inmunogénico<br>[25]
	CSP	*Pv*CSP-LSP	synthetic peptide<br>Montanide ISA 720/51	I		Colombia	immunogenic and safe<br>[26]
		VMP001<br>(Vivax malarial Protein 1)	Recombinant<br>AS01B	I	IIa	USA	Cellular and humoral response lacking sterilizing protection<br>NCT01157897<br>[27]
Blood stages	DBP	*Pv*DBP<br>ChAd63/MVA	ChAd63-*Pv*DBP / MVA-*Pv*DBP.	I		UK	Cellular and humoral response lacking sterilizing protection<br>NCT01816113<br>[28]

Table S3. Candidate vaccines for erythrocyte stages.

Antigen	Candidate	System/Platform	Phase I	Phase II	Population Children	Population Adults	Producer/Place	Result (Ref.)
	FMP2.1	Recombinant (3D7) AS01B or AS02A	Ia				USA	Immunogenic [29]
			Ia	IIa				No efficacy in natural infection NCT00385047 [30]
		Recombinant (3D7) AS02A	Ia				Mali	Immunogenic NCT00308061 [31]
			Ia					Immunogenic NCT00358332 [32]
				II				No efficacy in clinical malaria NCT00460525 [33-34]
AMA-1	PfAMA-1-FVO	Recombinant (FVO) Alhydrogel, AS02A or Montanide ISA 720	Ib				The Netherlands	Safe and immunogenic NCT00730782 [35]
			Ib					Functional antibodies NCT00730782 [36]
			Ia				Mali	Short-lasting antibodies NCT00431808 [37]
	AMA1-C1	Recombinant (FVO y 3D7) Alhydrogel	Ia				USA	Immunogenic [38]
			Ia				Mali	Functional antibodies NCT00343005 [39]
			Ia					Short-lasting antibodies NCT00341250 [40]
				II				No efficacy NCT00341250 [39-40]
		Recombinant (FVO y 3D7) Montanide® ISA 720	Ia				Australia	Immunogenic with local reactogenicity NCT00487916

						[40]
	AMA1 AdCh63/ MVA	Expressed in AdCh63 (first dose) and in MVA (boost)	I	IIa	Ucrania	Immunogenic NCT01095055 [38]
MSP1	FMP1	Recombinant (3D7) MSP1(42) AS02A	Ib		Kenya	Safe and immunogenic [41]
				IIb		No efficacy NCT00223990 [42]
			Ia		USA	Safe and immunogenic [43]
			Ib		Kenya	Safe and immunogenic [44]
	FMP010	Recombinant (FVO) MSP1(42) AS01B	Ia/b		USA/Kenya	Safe and immunogenic, heterologous cross-reactivity NCT00666380 [45]
	MSP1(42) C1 + CPG	Recombinante MSP1(42) (FVO y 3D7) Alhydrogel y CPG 7909	I		USA	Safe and immunogenic NCT00320658 [46]
	AdCh63-MSP1/ MVA-MSP1	Expressed in AdCh63 (first dose) and in MVA (boost)	Ia	IIa	Ucrania	Seroconversion, but poor immune response NCT01003314 [47]
MSP2	MSP2-C1	Montanide ISA 720	I		Australia	Reactogenic [48]
MSP3	MSP3 (LSP)	Alhydrogel	Ib		Burkina Faso	Tolerated and immunogenic NCT00452088 [49]
				IIb	Mali	NCT00652275
EBA-175	EBA 175 RII NG	Recombinant aluminum phosphate	I		USA	Safe and immunogenic NCT00347555 [50]
			Ia		Ghana	Safe and immunogenic NCT01026246 [51]
SERA-5	BK-SE36	Recombinant Alhydrogel	Ib		Uganda	Safe and immunogenic ISRCTN71619711 [52]

	SE36	Recombinant Aluminum salts	Ia		Japan	Safe and immunogenic ISRCTN78679862 [53]
GLURP	GLURP$_{85-213}$ (LSP) GMZ2	Alhydrogel / Montanide ISA 720	Ia		The Netherlands	Safe and immunogenic [54]
			**Multi-stage**			
AMA-1/ MSP1	PfCP2.9	Recombinant chymera AMA-1/MSP1$_{(19)}$ Montanide ISA 720	Ia		China	Antibodies lacking functional actiuvity NCT00284973 [55]
MSP1$_{(19)}$ + EBA-175	JAIVAC-1	Montanide ISA 720	Ia		India	Seroconversion but poor immune response CTRI/2010/091/000301 [56]
MSP1 MSP2 RESA	Combination B	Recombinant Montanide ISA 720		IIa	Australia	Do not stimulate immune response [57]
			Ib	IIb	Papua New Guinea	Parasitemia reduction; safe and immunogenic [58]
GLURP + MSP3	GMZ2	Alhydrogel	Ia/Ib		Germany	Safe and immunogenic NCT00397449 [57-58]
			Ia/Ib		Gabon	Induce antibodies and B-cell memory NCT00424944 [59] Safe and immunogenic NCT00703066 [60]

Table S4. Adjuvants in malaria vaccine candidates.

Adjuvant	Attributed Mechanism	Use and Applications	Reference
Aluminum salts (aluminum hydroxide, aluminum phosphate). Alhydrogel	Innate immune response activation by direct interaction with dendritic cells, or inducing release of cellular constituents that activate them	Hepatitis A, Hepatitis B, Diphtheria, Tetanus, Pertussis.	[61]
Muramyl dipeptide (MDP)	Activates various cell types (macrophages, polymorphonuclear leukocytes, mast cells, platelets, endothelial cells and fibroblasts, among others) and induces the secretion of a variety of cytokines	Anthrax (a disease caused by a gram-positive Bacillus anthracis), gram negative bacteria and bacterial toxins	[62]
Monophosphoryl Lipid A (MPL)	Stimulate antigen presentation to dendritic cells and macrophages, via toll-like receptors (TLRs) and promote effective uptake, processing and presentation of antigen to T-cells in draining lymph nodes	Cancer and tumor-associated antigens	[63]
Immune-stimulating complexes (ISCOMs)	Enhance class II MHC expression on APCs	9	[64]
Water-oil emmulsions	It is believed to act through the induction of local inflammation and increasing concentration and activation of APCs	VIH, malaria, breast cancer	[65]
Oil- water emmulsions	Innate inflammatory response, APC recruitment and activation, enhance persistence of antigen at the injection site, as well as presentation to immunocompetent cells	Seasonal flu	[66]
Montanide ISA 720 and 51 (water in oil)	Formation of a deposit at the injection site that enables slow antigen release, thereby stimulating antibody-producing cells	HIV and malaria	[67]
AddaVax: Oil Emulsion Adjuvant - MF59	Are in principle a squalene-based oil-in-water nano-emulsion system able to elicit both cellular (Th1) and humoral (Th2) immune responses. This class of adjuvants is believed to act through recruitment and activation of APC and stimulation of cytokines and chemokines production by macrophages and granulocytes	Veterinary and human applications	[68]
Virosome and liposome systems	Act mainly as a vehicle for the antigen delivery by their encapsulation, depending on their structure and other characteristics potentiating a humoral response	Hepatitis A, influenza, gam-negative bacteria	[69]
3-O-desacyl-4´-monophosphoryl lipid A (MPL)	Binds to TLR4 inducing humoral and cellular responses (predominantly Th1-based)	Hepatitis B	[70]
Saponin- QS21	Improves antigen presentation by APCs (predominant Th1 - Th2 responses)	Alzheimer	[71]
Adjuvant sysstems AS01. (AS03, S04 and AS015)	AS01 is a liposome-based vaccine adjuvant system containing two immunostimulants: 3-O-desacyl-4'-monophosphoryl lipid A (MPL) and the saponin QS-21 and is efficient at promoting CD4+ T cell-mediated immune responses	Human diseases	[72]
Immunostimulatory Sequences (ISS) for DNA	TLR agonists, induction of pro-inflammatory cytokines, amplification of adaptive immune response	Hepatitis B, malaria, HIV, cancer	[73]
Freund's Complete Adjuvant (FCA), contains mycobacterial walls / Freund's Incomplete Adjuvant (FIA)	Act by prolonging the life time of the injected antigen, promoting the proliferation and differentiation of CD4 + T lymphocytes. Th1 profile is induced in the presence of mycobacterium walls and in their absence stimulates a Th2 profile	Veterinary and experimental animal models	[74]

Table S5. Blocking transmission vaccine candidates.

Antigen	Candidate	System/Platform	Phase I	Phase II	Population Children	Population Adults	Producer/Place	Result/ ClinicalTrials.gov Identifier/ Reference
Pfs25	Pfs25	Recombinant<br>Montanide ISA-51	Ia				USA	Antibody levels related to transmission blockage. Systemic reactogenicity NCT00295581 [20]
	Pfs25M-EPA/Alhydrogel	Recombinant, conjugated to exoprotein A (EPA)	Ia				USA	Dose-dependent increase in immunogenicity induces transmission blockade. NCT01434381 [21]
			Ia				Mali	Antibody titers correlated with functional activity NCT01867463 [75]
Pfs25/230	Pfs25M- EPA/Alhydrogel Pfs230D1M-EPA/Alhydrogel	Recombinant conjugated to EPA	Ia				EE. UU	In progress NCT02334462 [76]
		AS01	I				Malí	In progress NCT02942277 [77]
Pfs47	Antibody targeting [78]							

References

1. Ewer, K.; O'Hara, G.; Duncan, C.; Collins, K.; Sheehy, S.; Reyes-Sandoval, A.; Goodman, A.; Edwards, N.; Elias, S.; Halstead, F.; et al. Protective CD8+ T-cell immunity to human malaria induced by chimpanzee adenovirus-MVA immunisation. Nat. Commun. 2013, 4, 2836, doi:10.1038/ncomms3836.
2. Hodgson, S.; Ewer, K.; Bliss, C.; Edwards, N.; Rampling, E.; Anagnostou, N.; De Barra, E.; Havelock, T.; Bowyer, G.; Poulton, I.; et al. Evaluation of the Efficacy of ChAd63-MVA Vectored Vaccines Expressing Circumsporozoite Protein and ME-TRAP Against Controlled Human Malaria Infection in Malaria-Naive Individuals. J. Infect. Dis. 2015, 211, 1076–1086, doi:10.1093/infdis/jiu579.
3. Afolabi, M.; Tiono, A.; Uche, J., A.; Yaro, J.; Drammeh, A.; Nébié, I.; Bliss, C.; Hodgson, S.; Anagnostou, N.; Sanou, G.; et al. Safety and Immunogenicity of ChAd63 and MVA ME-TRAP in West African Children and Infants. Mol. Ther. 2016, 24, 1470–1477, doi:10.1038/mt.2016.83.
4. Ogwang, C.; Afolabi, M.; Kimani, D.; Jagne, YJ.; Sheehy, SH.; Bliss, CM.; Duncan, CJ.; Collins, KA.; Garcia, MA.; Kimani, E.; et al. Safety and immunogenicity of heterologous prime-boost immunisation with Plasmodium falciparum malaria candidate vaccines, ChAd63 ME-TRAP and MVA ME-TRAP, in healthy Gambian and Kenyan adults. PLoS One 2013, 8, e57726. doi: 10.1371/journal.pone.0057726.
5. Pirahmadi, S.; Zakeri, S.; Mehrizi, A.; Djadid, N.; Raz, A.; Sani, J. Combining Monophosphoryl Lipid A (MPL), CpG Oligodeoxynucleotide (ODN), and QS-21 Adjuvants Induces Strong and Persistent Functional Antibodies and T Cell Responses against Cell-Traversal Protein for Ookinetes and Sporozoites (CelTOS) of Plasmodium falciparum in BALB/c Mice. Infect Immun. 2019, 87, e00911–18, doi:10.1128/IAI.00911-18.
6. Prieur, E.; Druilhe, P. The malaria candidate vaccine liver stage antigen-3 is highly conserved in Plasmodium falciparum isolates from diverse geographical areas. Malar. J. 2009, 8, 247, doi:10.1186/1475-2875-8-247.
7. Hoffman, S.; Vekemans, J.; Richie, T.; Duffy, P. The march toward malaria vaccines. Vaccine 2015, 33, D13–D23, doi:10.1016/j.vaccine.2015.07.091.
8. Creech, B.; Dekker, C.; Ho, D.; Phillips, S.; Mackey, S.; Murray-Krezan, C.; Grazia, M.; Hendriks, J.; Brown, V.; Dally, L.; et al. Randomized, placebo-controlled trial to assess the safety and immunogenicity of an adenovirus type 35-based circumsporozoite malaria vaccine in healthy adults. Hum. Vaccin Immunother. 2013, 2548–2557, doi:10.4161/hv.26038.
9. Ouédraogo, A.; Tiono, A.; Kargougou, D.; Yaro, J.; Ouédraogo, E.; Kaboré, Y.; Kangoye, D.; Bougouma, E.; Gansane, A.; Henri, N.; et al. A phase 1b randomized, controlled, double-blinded dosage-escalation trial to evaluate the safety, reactogenicity and immunogenicity of an adenovirus type 35 based circumsporozoite malaria vaccine in Burkinabe healthy adults 18 to 45 years of age. PLoS ONE 2013, 11, e78679, doi:10.1371/journal.pone.0078679.
10. Seder, R.; Chang, L.; Enama, M.; Zephir, K.; Sarwar, U.; Gordon, I.; Holman, L.; James, E.; Billingsley, P.; Gunasekera, A., et al. Protection against malaria by intravenous immunization with a nonreplicating sporozoite vaccine. Science 2013, 341, 1359–1365, doi:10.1126/science.1241800.
11. Lyke, K.; Singer, A.; Berry, A.; Reyes, S.; Chakravarty, S.; James, E.; Billingsley, PF.; Gunasekera, A.; Manoj, A.; Murshedkar, T.; et al. II Study Team. Multidose Priming and Delayed Boosting Improve PfSPZ Vaccine Efficacy against Heterologous P. falciparum Controlled Human Malaria Infection. Clin. Infect. Dis 2020, ciaa1294, doi: 10.1093/cid/ciaa1294.
12. Lyke, K.; Ishizukab, A.; Berrya, A.; Chakravartyc, S.; DeZure, A.; Enama, M.; James, E.; Billingsley, PF.; Gunasekera, A.; Mano, A.; et al. Attenuated PfSPZ Vaccine induces strain-transcending T cells and durable protection against heterologous controlled human malaria infection. Proc. Natl. Acad. Sci. USA 2017, 114, 2711–2716, doi:10.1073/pnas.1615324114.
13. Zaidi, I.; Diallo.; Conteh, S.; Robbins, Y.; Kolasny, J.; Orr-Gonzalez, S.; Carter, D.; Butler, B.; Lambert, L.; Brickley, E.; et al. γδ T Cells Are Required for the Induction of Sterile Immunity during Irradiated Sporozoite Vaccinations. J Immunol 2017, 199, 3781-3788, doi: 10.4049/jimmunol.1700314.
14. Roestenberg, M.; McCall, M.; Hopman, J.; Wiersma, J.; Luty, A.; Gemert, G.; Vegte-Bolmer, M.; Schaijk, B.; Teelen, K.; Arens, T. et al. Protection against a malaria challenge by sporozoite inoculation. N. Engl. J. Med. 2009, 361, 468–477, doi:10.1056/NEJMoa0805832.

15. Roestenberg, M.; Teirlinck, A.; McCall, M.; Teelen, K.; Makamdop, K.; Wiersma, J.; Arens, T.; Beckers, P.; Gemert, G.; Vegte-Bolmer, M.; et al. Long-term protection against malaria after experimental sporozoite inoculation: An open-label follow-up study. Lancet 2011, 377, 1770–1776, doi:10.1016/S0140-673660360-7.
16. Achan, J.; Reuling, IJ.; Yap, XZ.; Dabira, E.; Ahmad, A.; Cox, M.; Nwakanma, D.; Tetteh, K.; Wu, L.; Bastiaens, G.; et al. Serologic Markers of Previous Malaria Exposure and Functional Antibodies Inhibiting Parasite Growth Are Associated With Parasite Kinetics Following a Plasmodium falciparum Controlled Human Infection. Clin Infect Dis 2020, 70, 2544-2552, doi: 10.1093/cid/ciz740.
17. Spring, M.; Murphy, J.; Nielsen, R.; Dowler, M.; Bennett, J.; Zarling, S.; Williams, J.; De la Vega, P.; Ware, L.; Komisar, J. et al. First-in-human evaluation of genetically attenuated Plasmodium falciparum sporozoites administered by bite of Anopheles mosquitoes to adult volunteers. Vaccine 2013, 31, 4975–4983, doi:10.1016/j.vaccine.2013.08.007.
18. Murphy, S.; Duke, E.; Shipman, K.; Jensen, R.; Fong, Y.; Ferguson, S.; Janes, H.; Gillespie, K.; Seilie, A.; Hanron, A.; et al. A Randomized Trial Evaluating the Prophylactic Activity of DSM265 Against Preerythrocytic Plasmodium falciparum Infection During Controlled Human Malarial Infection by Mosquito Bites and Direct Venous Inoculation. J Infect Dis 2018, 217, 693-702, doi: 10.1093/infdis/jix613.
19. van Dijk MR, Douradinha B, Franke-Fayard B, Heussler V, van Dooren MW, van Schaijk B, van Gemert GJ, Sauerwein RW, Mota MM, Waters AP, Janse CJ. Genetically attenuated, P36p-deficient malarial sporozoites induce protective immunity and apoptosis of infected liver cells. Proc Natl Acad Sci U S A. 2005 Aug 23;102(34):12194-9. doi: 10.1073/pnas.0500925102.
20. Shimp, R.; Rowe, C.; Reiter, K.; Chen, B.; Nguyen, V.; Aebig J, Rausch, K.; Kumar K, Wu Y, Jin, A.; et al. Development of a Pfs25-EPA malaria transmission blocking vaccine as a chemically conjugated nanoparticle. Vaccine 2013, 31, 2954-62, doi: 10.1016/j.vaccine.2013.04.034.
21. Zhu, D.; Wu, Y.; McClellan, H.; Dai, W.; Rausch, K.; Jones, D.; Aebig, J.; Barnafo, E.; Butler, B.; Lambert, L.; et al. Accelerated and long-term stability study of Pfs25-EPA conjugates adjuvanted with Alhydrogel. Vaccine 2017, 35, 3232-3238, doi: 10.1016/j.vaccine.2017.04.067.
22. Da, D.; Dixit, S.; Sattabonkot, J.; Mu, J.; Abate, L.; Ramineni, B.; Ouedraogo, JB.; MacDonald, N.; Fay, MP.; Su X.; et al. Anti-Pfs25 human plasma reduces transmission of Plasmodium falciparum isolates that have diverse genetic backgrounds. Infect Immun 2013, 81, 1984-1989, doi: 10.1128/IAI.00016-13.
23. Blagborough, A.; Yoshida, S., Sattabongkot, J.; Tsuboi, T.; Sinden, R. Intranasal and intramuscular immunization with Baculovirus Dual Expression System-based Pvs25 vaccine substantially blocks Plasmodium vivax transmission. Vaccine 2010, 28, 6014-20, doi: 10.1016/j.vaccine.2010.06.100.
24. Rawlinson, T.; Barber, N.; Mohring, F.; Cho, JS.; Kosaisavee, V.; Gérard, SF.; Alanine, D.; Labbé, G.; Elias, S.; Silk, S.; et al. Structural basis for inhibition of Plasmodium vivax invasion by a broadly neutralizing vaccine-induced human antibody. Nat Microbiol 2019, 4, 1497-1507, doi: 10.1038/s41564-019-0462-1.
25. Bauza, K.; Malinauskas, T.; Pfander, C.; Anar, B.; Jones, E.; Billker, O.; Hill, A.; Reyes-Sandoval, A. Efficacy of a Plasmodium vivax malaria vaccine using ChAd63 and modified vaccinia Ankara expressing thrombospondin-related anonymous protein as assessed with transgenic Plasmodium berghei parasites. Infect. Immun. 2014, 82, 1277–1286, doi:10.1128/IAI.01187-13.
26. Bennett, J.; Yadava, A.; Tosh, D.; Sattabongkot, J.; Komisar, J.; Ware, L.A.; McCarthy, W.; Cowden, J.; Regules, J.; Spring, M.; Paolino, K.; et al. Phase 1/2a Trial of Plasmodium vivax Malaria Vaccine Candidate VMP001/AS01B in Malaria-Naive Adults: Safety, Immunogenicity, and Efficacy. PLoS Negl. Trop. Dis. 2016, 10, e0004423, doi:10.1371/journal.pntd.0004423.
27. Herrera, S.; Fernández, O.; Vera, O.; Cárdenas, W.; Ramírez, O; Palacios, R.; Chen-Mok, M.; Corradin, G.; Arévalo-Herrera, M. Phase I safety and immunogenicity trial of Plasmodium vivax CS-derived long synthetic peptides adjuvanted with Montanide ISA 720 or ISA 51. Am. J. Trop. Med. Hyg. 2011, 84, 12–20, doi:10.4269/ajtmh.2011.09-0516.
28. Payne RO, Silk SE, Elias SC, Milne KH, Rawlinson TA, Llewellyn D, Shakri AR, Jin J, Labbé GM, Edwards NJ, Poulton ID, Roberts R, Farid R, Jørgensen T, Alanine DG, de Cassan SC, Higgins MK, Otto TD, McCarthy JS, de Jongh WA, Nicosia A, Moyle S, Hill AV, Berrie E, Chitnis CE, Lawrie AM, Draper SJ. Human vaccination against Plasmodium vivax Duffy-binding protein induces strain-transcending antibodies. JCI Insight. 2017 Jun 15;2(12):e93683. doi: 10.1172/jci.insight.93683.

29. Polhemus, M.; Magill, A.; Cummings, J.; Kent, K.; Ockenhouse, C.; Lanar, D.; Dutta, S.; Barbosa, A.; Soisson, L.; Diggs, C.; et al. Phase I dose escalation safety and immunogenicity trial of Plasmodium falciparum apical membrane protein (AMA-1) FMP2.1, adjuvanted with AS02A, in malaria-naïve adults at the Walter Reed Army Institute of Research. Vaccine 2007, 25, 4203–4212, doi:10.1016/j.vaccine.2007.03.012.
30. Thera, M.; Doumbo, O.; Coulibaly, D., Diallo, D.; Kone, A.; Guindo, A.; Traore, K.; Dicko, A.; Sagara, I.; Sissoko, M.; et al. Safety and immunogenicity of an AMA-1 malaria vaccine in Malian adults: Results of a phase 1 randomized controlled trial. PLoS ONE 2008, 3, e1465, doi:10.1371/journal.pone.0001465.
31. Thera, M.; Doumbo, O., Coulibaly, D.; Laurens, M.; Kone, A; Guindo, A.; Traore, K.; Sissoko, M.; Diallo, D.; Diarra, I.; et al. Safety and Immunogenicity of an AMA1 Malaria Vaccine in Malian Children: Results of a Phase 1 Randomized Controlled Trial. PLoS ONE 2010, 5, e9041, doi:10.1371/journal.pone.0009041.
32. Thera, M.; Doumbo, O.; Coulibaly, D.; Laurens, M.; Ouattara, A.; Kone, A.; Guindo, A.; Traore, K.; Traore, I.; Kouriba, B.; et al. A field trial to assess a blood-stage malaria vaccine. N. Engl. J. Med. 2011, 365, 1004–1013, doi:10.1056/NEJMoa1008115.
33. Dicko, A.; Diemert, D.; Sagara, I.; Sogoba, M.; Niambele, M.; Assadou, M.; Guindo, O.; Kamate, B.; Baby, M.; Sissoko, M.; et al. Impact of a Plasmodium falciparum AMA1 vaccine on antibody responses in adult Malians. PLoS ONE 2007, 2, e1045, doi:10.1371/journal.pone.0001045.
34. Dicko, A.; Sagara, I.; Ellis, R.; Miura, K.; Guindo, O.; Kamate, B.; Kamate, B.; Sogoba, M.; Niambelé, M.; Sissoko, M.; Baby, M.; et al. Phase 1 study of a combination AMA1 blood stage malaria vaccine in Malian children. PLoS ONE 2008, 3, e1563, doi:10.1371/journal.pone.0001563.
35. Thera, M.; Coulibaly, D.; Kone, A.; Guindo, A.; Traore, K.; Sall, A.; Diarra, I.; Daou, M.; Traore, I.; Tolo, Y.; et al. Phase 1 randomized controlled trial to evaluate the safety and immunogenicity of recombinant Pichia pastoris-expressed Plasmodium falciparum apical membrane antigen 1 (PfAMA1-FVO [25-545]) in healthy Malian adults in Bandiagara. Malar J 2016, 15, 442, doi: 10.1186/s12936-016-1466-4.
36. Pierce, M.; Ellis, R.; Martin, L.; Malkin, E.; Tierney, E.; Miura, K.; Fay, M.; Marjason, J.; Elliott, S.; Mullen, G.; et al. Phase 1 Safety and Immunogenicity Trial of the Plasmodium falciparum Blood-Stage Malaria Vaccine AMA1-C1/ISA720 in Australian Adults. Vaccine 2010, 28, 2236–2242, doi:10.1016/j.vaccine.2009.12.049.
37. Laurens, M.; Thera, M.; Coulibaly, D.; Ouattara, A.; Kone, A.; Guindo, A.; Traore, K.; Traore, I.; Kouriba, B.; Diallo, D.; et al. Extended Safety, Immunogenicity and Efficacy of a Blood-Stage Malaria Vaccine in Malian Children: 24-Month Follow-Up of a Randomized, Double-Blinded Phase 2 Trial. PLoS ONE 2013, 8, e79323, doi:10.1371/journal.pone.0079323.
38. Roestenberg, M.; Remarque, E.; de Jonge, E.; Hermsen, R.; Blythman, H.; Leroy, L.; Imoukhuede, E.; Jepsen, S.; Ofori-Anyinam, O.; Faber, F.; et al. Safety and immunogenicity of a recombinant Plasmodium falciparum AMA1 malaria vaccine adjuvanted with Alhydrogel, Montanide ISA 720 or AS02. PLoS ONE 2008, 3, e3960, doi:10.1371/journal.pone.0003960.
39. Remarque, E.; Roestenberg, M.; Younis, S.; Walraven, V.; van der Werff, N; Faber, B.; Leroy, O.; Sauerwein, S.; Kocken, C.; Thomas, A. Humoral Immune Responses to a Single Allele PfAMA1 Vaccine in Healthy Malaria-Naıve Adults. PLoS ONE 2012, 7, e38898, doi:10.1371/journal.pone.0038898.
40. Thera, M.; Coulibaly, D.; Kone, A., Guindo, A.; Traore, K.; Sall, A.; Diarra, I.; Daou, M.; Traore, I.; Tolo, Y.; et al. Phase 1 randomized controlled trial to evaluate the safety and immunogenicity of recombinant Pichia pastoris-expressed Plasmodium falciparum apical membrane antigen 1 (PfAMA1-FVO [25–545]) in healthy Malian adults in Bandiagara. Malar. J. 2016, 15, 442, doi:10.1186/s12936-016-1466-4.
41. Withers, M.; McKinney, D.; Ogutu, B.; Waitumbi, J.; Milman, J.; Apollo, O.; Allen, O.; Tucker, K.; Soisson, L.; Diggs, C.; et al. Safety and Reactogenicity of an MSP-1 Malaria Vaccine Candidate: A Randomized Phase Ib Dose-Escalation Trial in Kenyan Children. PLoS Clin. Trials. 2006, 1, e32, doi:10.1371/journal.pctr.0010032.
42. Ogutu, B.; Apollo, O.; McKinney, D.; Okoth, W.; Siangla, J.; Dubovsky, F.; Tucker, K.; Waitumbi, J.; Diggs, C.; Wittes, J.; et al. Blood Stage Malaria Vaccine Eliciting High Antigen-Specific Antibody Concentrations Confers No Protection to Young Children in Western Kenya. PLoS ONE 2009, 4, e4708, doi:10.1371/journal.pctr.0010032.

43. Ockenhouse, C.; Angov, E.; Kester, K.; Diggs, C.; Soisson, L.; Cummings, J.; Stewart, A.; Palmer, D.; Mahajan, B.; Krzych, U.; et al. Phase I safety and immunogenicity trial of FMP1/AS02A, a Plasmodium falciparum MSP-1 asexual blood stage vaccine. Vaccine 2006, 24, 3009–3017, doi:10.1016/j.vaccine.2005.11.028.
44. Stoute, J.; Gombe, J.; Withers, M.; Siangla, J.; McKinney, D.; Onyango, M:; Cummings, J.; Milman, J.; Tucker, K.; Soisson, L.; et al. Phase 1 randomized double-blind safety and immunogenicity trial of Plasmodium falciparum malaria merozoite surface protein FMP1 vaccine, adjuvanted with AS02A, in adults in western Kenya. Vaccine 2007, 25, 176–184, doi:10.1016/j.vaccine.2005.11.037.
45. Otsyula, N.; Angov, E.; Bergmann-Leitner, E.; Koech, M.; Khan, F.; Bennett, J.; Otieno, L.; Cummings, J.; Andagalu, B.; Tosh, D.; et al. Results from tandem Phase 1 studies evaluating the safety, reactogenicity and immunogenicity of the vaccine candidate antigen Plasmodium falciparum FVO merozoite surface protein-1 (MSP142) administered intramuscularly with adjuvant system AS01. Malar. J. 2013, 12, 29, doi:10.1186/1475-2875-12-29.
46. Ellis, R.; Martin, L.; Shaffer, D.; Long, C.; Miura, K.; Fay, M.; Narum, D.; Zhu, D.; Mullen, G.; Mahanty, S.; et al. Phase 1 trial of the Plasmodium falciparum blood stage vaccine MSP1-C1/Alhydrogel with and without CPG 7909 in malaria naïve adults. PLoS ONE 2010, 5, e8787, doi:10.1371/journal.pone.0008787.
47. Sheehy, S.; Duncan, C.; Elias, S.; Collins, K.; Ewer, K.; Spencer, A.; Williams, A.; Halstead, F.; Moretz, S.; Miura, K.; et al. Phase Ia clinical evaluation of the Plasmodium falciparum blood-stage antigen MSP1 in ChAd63 and MVA vaccine vectors. Mol. Ther. 2011, 19, 2269–2276, doi:10.1038/mt.2011.176.
48. McCarthy JS, Marjason J, Elliott S, Fahey P, Bang G, Malkin E, Tierney E, Aked-Hurditch H, Adda C, Cross N, Richards JS, Fowkes FJ, Boyle MJ, Long C, Druilhe P, Beeson JG, Anders RF. A phase 1 trial of MSP2-C1, a blood-stage malaria vaccine containing 2 isoforms of MSP2 formulated with Montanide ISA 720. PLoS One. 2011;6(9):e24413. doi: 10.1371/journal.pone.0024413.
49. Sirima, S.; Tiono, A.; Ouédraogo, A.; Diarra, A.; Ouédraogo, A.; Yaro, JP.; Ouédraogo, E.; Gansané, A.; Bougouma, E.; Konaté, A.; et al. Safety and immunogenicity of the malaria vaccine candidate MSP3 long synthetic peptide in 12–24 months-old Burkinabe children. PLoS ONE 2009, 4, e7549, doi:10.1371/journal.pone.0007549.
50. El Sahly, H.; Patel, S.; Atmar, R.; Lanford, T.; Dube, T.; Thompson, D.; Sim, B.; Long, C.; Keitel, W. The safety and immunogenicity of recombinant EBA 175-RII NG malaria vaccine in healthy adults living in a non-endemic area. Clin. Vaccine Immunol. 2010, 1552–1559, doi:10.1128/CVI.00082-10.
51. Kusi, K.; Manu, E.; Manful, T.; Kyei-Baafour, E.; Dickson, E.; Amponsah, J.; Remarque, E.; Faber, B.; Kocken, C.; Dodoo D, et al. Variations in the quality of malaria-specific antibodies with transmission intensity in a seasonal malaria transmission area of Northern Ghana. PLoS One 2017, 12, e0185303, doi: 10.1371/journal.pone.018530.
52. Ezoe, S.; Palacpac, N.; Tetsutani, K.; Yamamoto, K.; Okada, K.; Taira, M.; Nishida, S.; Hirata, H.; Ogata, A.; Yamada T.; et al. First-in-human randomised trial and follow-up study of Plasmodium falciparum blood-stage malaria vaccine BK-SE36 with CpG-ODN(K3). Vaccine 2020, 38, 7246-7257, doi: 10.1016/j.vaccine.2020.09.056.
53. Horii, T.; Shirai, H.; Jie, L.; Palacpac, N.; Tougan, T.; Hato, M.; Ohta, M.; Bobogare, A.; Arakaki, N.; Matsumoto, Y.; et al. Evidences of protection against blood-stage infection of Plasmodium falciparum by the novel protein vaccine SE36. Parasitol. Int. 2010, 59, 380–386, doi:10.1016/j.parint.2010.05.002.
54. Turner, L.; Wang, C.; Lavstsen, T.; Mwakalinga, S.; Sauerwein, R.; Hermsen, C.; Theander, T. Antibodies against PfEMP1, RIFIN, MSP3 and GLURP are acquired during controlled Plasmodium falciparum malaria infections in naive volunteers. PLoS One 2011, 6, e29025, doi: 10.1371/journal.pone.0029025.
55. Malkin, E.; Hu, J.; Li, Z.; Chen, Z., Bi, X.; Reed, Z.; Dubovsky, F.; Liu, J.; Wang, O.; Pan, X.; et al. A phase 1 trial of PfCP2.9: An AMA1/ MSP1 chimeric recombinant protein vaccine for Plasmodium falciparum malaria. Vaccine 2008, 26, 6864–6873, doi:10.1016/j.vaccine.2008.09.081.
56. Chitnis, C.; Mukherjee, P.; Mehta, S.; Yazdani, S.; Dhawan, S.; Shakri, A.; Bhardwaj, R.; Gupta, P.; Hans, D.; Mazumdar, S.; et al. Phase I Clinical Trial of a Recombinant Blood Stage Vaccine Candidate for Plasmodium falciparum Malaria Based on MSP1 and EBA175. PLoS ONE 2015, 10, e0117820, doi:10.1371/journal.pone.0117820.

57. Lawrence, G.; Cheng, Q.; Reed, C.; Taylor, D.; Stowers, A.; Cloonan, N.; Rzepczyk, C.; Smillie, A.; Anderson, K.; Pombo, D.; et al. Effect of vaccination with 3 recombinant asexual-stage malaria antigens on initial growth rates of Plasmodium falciparum in non-immune volunteers. Vaccine 2000, 18, 1925–1931, doi:10.1016/s0264-410x(99)00444-2.
58. Genton, B., Betuela, I.; Felger, I.; Al-Yaman, F.; Anders, R; Saul, A.; Rare, L.; Baisor, M.; Lorry, K.; Brown, G.; et al. A recombinant blood-stage malaria vaccine reduces Plasmodium falciparum density and exerts selective pressure on parasite populations in a phase 1-2b trial in Papua New Guinea. J. Infect. Dis. 2002, 185, 820–827, doi:10.1086/339342.
59. Esen, M.; Kremsner, P.; Schleucher, R.; Gässler, M.; Imoukhuede, E.; Imbault, N.; Leroy, O.; Jepsen, S.; Knudsen, B.; Schumm, M.; et al. Safety and immunogenicity of GMZ2-a MSP3-GLURP fusion protein malaria vaccine candidate. Vaccine 2009, 27, 6862–6868, doi:10.1016/j.vaccine.2009.09.011.
60. Mordmüller, B.; Szywon, K.; Greutelaers, B.; Esen, M.; Mewono, L.; Treut, C.; Mürbeth, R.; Chilengi, R.; Noor, R.; Kilama, W.; et al. Safety and immunogenicity of the malaria vaccine candidate GMZ2 in malaria-exposed, adult individuals from Lambaréné, Gabon. Vaccine 2010, 28, 6698–6703, doi:10.1016/j.vaccine.2010.07.085.
61. Baylor, N.; Egan, W.; Richman, P. Aluminum salts in vaccines US perspective. Vaccine 2002, 20, S18–S23, doi:10.1016/s0264-410x(02)00166-4.
62. Ogawa, C.; Liu, Y.J.; Kobayashi, K.S. Muramyl dipeptide and its derivatives: Peptide adjuvant in immunological disorders and cancer therapy. Curr. Bioact. Compd. 2011, 7, 180–197, doi:10.2174/157340711796817913.
63. Cluff, C. Advances in Experimental Medicine and Biology. In Lipid A in Cancer Therapy; Jeannin, J., Ed.; Springer-Verlag: New York, NY, USA, 2010; Volume 667.doi:10.1007/978-1-4419-1603-7.
64. Encyclopedia of Immunology. In Second Edition eBook; Peter, Delves, J., Ed.; Elsevier Inc: Amsterdam, The Netherlands, 1998; ISBN: 9780080547879.
65. Garçon, N.; Friede, M. Plotkin’s Vaccines (Seventh Edition). In Evolution of Adjuvants Across the Centuries; Stanley, A., Plotkin, Walter, A., Paul, A., Kathryn, M., Eds.; Elsevier: Amsterdam, The Netherlands, 2018; pp. 61–74.e4. ISBN: 9780323357616, doi:10.1016/B978-0-323-35761-6.00006-7.
66. Haensler, J. Manufacture of Oil-in-Water Emulsion Adjuvants. Methods Mol. Biol. 2017, 1494, 165–180, doi:10.1007/978-1-4939-6445-1_12.
67. Aucouturier, J.; Dupuis, L.; Deville, S.; Ascarateil, S.; Ganne, V. Montanide ISA 720 and 51: A new generation of water in oil emulsions as adjuvants for human vaccines. Expert Rev. Vaccines 2002, 1, 111–118, doi:10.1586/14760584.1.1.111.
68. Calabro, S.; Tritto, E.; Pezzotti, A.; Taccone, M.; Muzzi, A.; Bertholet, S.; De Gregorio, E.; O'Hagan, D.; Baudner, B.; Seubert, A. The adjuvant effect of MF59 is due to the oil-in-water emulsion formulation, none of the individual components induce a comparable adjuvant effect. Vaccine 2013, 31, 3363–3369, doi:10.1016/j.vaccine.2013.05.007.
69. Medical Applications of Liposomes; Lasic, D.D., Papahadjopoulos, D., Eds.; Elsevier Science: Amsterdam, The Netherlands, 1998.
70. Michaud, JP.; Hallé, M.; Lampron, A.; Thériault, P.; Préfontaine, P.; Filali, M.; Tribout-Jover, P.; Lanteigne, AM.; Jodoin, R.; Cluff C.; et al. Toll-like receptor 4 stimulation with the detoxified ligand monophosphoryl lipid A improves Alzheimer's disease-related pathology. Proc. Natl. Acad. Sci. USA. 2013, 110, 1941-1946, doi: 10.1073/pnas.1215165110.
71. Zhu, D.; Tuo, W. QS-21: A Potent Vaccine Adjuvant. Nat. Prod. Chem. Res. 2016, 3, e113, doi:10.4172/2329-6836.1000e113.
72. Didierlaurent, A.; Laupèze, B.; Di Pasquale, A.; Hergli, N.; Collignon, C.; Garçon, N. Adjuvant system AS01: Helping to overcome the challenges of modern vaccines. Expert Rev. Vaccines 2017, 16, 55–63, doi:10.1080/14760584.2016.1213632.
73. Higgins, D.; Marshall, J.; Traquina, P.; Van Nest, G.; Livingston, B. Immunostimulatory DNA as a vaccine adjuvant. Expert Rev. Vaccines 2007, 6, 747–759, doi:10.1586/14760584.6.5.747.
74. Hanlon, K.E.; Vanderah, T.W. Constitutive activity at the cannabinoid CB(1) receptor and behavioral responses. Methods Enzymol. 2010, 484, 3–30, doi:10.1016/B978-0-12-381298-8.00001-0.
75. Huang WC, Deng B, Mabrouk MT, Seffouh A, Ortega J, Long C, Miura K, Wu Y, Lovell JF. Particle-based, Pfs230 and Pfs25 immunization is effective, but not improved by duplexing at fixed total antigen dose. Malar J. 2020 Aug 28;19(1):309. doi: 10.1186/s12936-020-03368-5.

76. Singh K, Burkhardt M, Nakuchima S, Herrera R, Muratova O, Gittis AG, Kelnhofer E, Reiter K, Smelkinson M, Veltri D, Swihart BJ, Shimp R Jr, Nguyen V, Zhang B, MacDonald NJ, Duffy PE, Garboczi DN, Narum DL. Structure and function of a malaria transmission blocking vaccine targeting Pfs230 and Pfs230-Pfs48/45 proteins. Commun Biol. 2020 Jul 24;3(1):395. doi: 10.1038/s42003-020-01123-9.
77. Scaria PV, Chen BB, Rowe CG, Alani N, Muratova OV, Barnafo EK, Lambert LE, Zaidi IU, Lees A, Rausch KM, Narum DL, Duffy PE. Comparison of carrier proteins to conjugate malaria transmission blocking vaccine antigens, Pfs25 and Pfs230. Vaccine. 2020 Jul 22;38(34):5480-5489. doi: 10.1016/j.vaccine.2020.06.018.
78. Canepa, G.; Molina-Cruz, A.; Yenkoidiok-Douti, L.; Burkhardt, M.; Peng, F.; Narum, D.; Boulanger, M.; Valenzuela, J., et al. Antibody targeting of a specific region of Pfs47 blocks Plasmodium falciparum malaria transmission. NPJ Vaccines 2018, 3, 26, doi:10.1038/s41541-018-0065-5.
